# 

**Published:** 1903-02-14

**Authors:** 


### NOTICE TO CORRESPONDENTS.

All MSS., Letters, Books for Review, and other matters intended for the Editor should be addressed The Editor, "The Hospital," The Hospital Building, 28 & 29 Southampton Street, Strand, W.C.

The Editor cannot undertake to return rejected MS., even when accompanied by stamped directed envelope.

**Notice to Advertisers and Subscribers.**

All Advertisements, Orders for Copies of The Hospital, and Business Communications should be addressed to **THE MANAGER, (Not to the Editor)**, The Hospital, 28 & 29 Southampton Street, Strand, London, W.C.

The Cost of Subscription, post free, is as follows.—Per week, 2½d.; per quarter, 2s. 8d.; per half-year, 5s. 3d.; per annum, 10s. 6d. Foreign:—Per annum, 16s. 2d., payable, in all cases, in advance.

**NOTICE.—Vol. XXXII. of "The Hospital" (April to September 1902) in handsome cloth binding is now on sale at the office of this Journal, price, 7s. 6d. Cases for binding the Numbers contained in this Volume 1s. 6d. Index to Vol. XXXII., 2½d., post free.**

**The Monthly Part for February is now ready. Price 6d., by post 8d.**

### THE STUDENT OF MEDICINE.

### APPOINTMENTS.

**Ernest H. Birchall**, L.R.C.P., L.R.C.S.Edin., appointed Medical Officer of the Aston Union Workhouse. **J. M. Crocker**, M.R.C.S., L.R.C.P.Lond., appointed District Medical Officer of the Keighley Union. **A. W. W. Dowding**, M.D.Durh., M.R.C.P. Edin., appointed District Medical Officer of the St. Columb Major Union. **J. G. Emanuel**, M.D., B.S., B.Sc., M.R.C.P.Lond., appointed Second Physician for Outpatients at the Queen's Hospital, Birmingham. **G. E. A. Evans**, M.R.C.S., L.R.C.P., appointed District Medical Officer of the Honiton Union. **Hy. Hutchinson**, L.R.C.P.I., L.F.P.S.Glas., L.S.A.Lond., appointed Surgeon to the London and India Docks Employés for the Districts of East Ham, Upton, and Manor Park. **W. E. Jolliffe**, L.R.C.P., L.R.C.S.Irel., appointed Resident Assistant Medical Officer to the Southampton Union. **C. Howarth Lee**, M.B., Ch.B.Vict., appointed Resident Medical Officer to St. Mary's Hospital, Manchester. **R. Prynne Marshall**, M.R.C.S.Eng., L.R.C.P.Lond., appointed Medical Officer to the Third District of the St. Olave's Union, and Certifying Surgeon to the Bermondsey Borough Council. **H. Merrall**, M.B., Ch.B.Vict., appointed Medical Officer of the St. Anne's District of the Fylde Union. **E. V. Perry**, M.R.C.S., L.R.C.P., appointed District Medical Officer of the Mitford and Launditch Union. **W. R. Reith**, M.D., C.M.Aberd., appointed Medical Officer for the Erdington District of the Aston Union. **James E. H. Sawyer**, M.A., M.B., B.Ch.Oxon., appointed Honorary Physician and Referee for Birmingham and the Midland Counties Home for Incurables, Leamington. **G. N. Stewart**, M.A., D.Sc., M.D.Edin., D.P.H.Camb., Professor of Physiology, Western Reserve University, Cleveland, U.S.A., appointed Professor of Physiology in the University of Chicago.

**"The Hospital" Institutional Library.**

"Hospitals and Asylums of the World." 4 Vols., with a Portfolio of Plans, complete £12 12s.
"Burdett's Hospitals and Charities, 1902." 5s.
"Cottage Hospitals, General, Fever, and Convalescent." 10s. 6d.
"Hospital Expenditure: The Commissariat." 2s. 6d.
"A Legal Handbook for the Use of Hospital Authorities." 2s. 6d.
"The Cottage Hospital Case Book or Register of Patients." 10s. and 12s. 6d.
"The Furnishing and Appliances of a Cottage Hospital." 6d.
All these are published by the SCIENTIFIC PRESS, Ltd., and may be obtained through any bookseller, or direct from the publishers, 28 and 29 Southampton Street, Strand, London, W.C.

### Useful Books for Nurses' Libraries.

**NURSING.**

**THE NURSING PROFESSION: HOW AND WHERE TO TRAIN.**

Being a Guide to Training for the Calling of a Nurse.

Edited by Sir Henry Burdett, K.C.B.

Crown 8vo., 2/- net, 2/4 post free.

**A HANDBOOK FOR NURSES.**

By J. K. Watson, M.D., M.B., C.M.

New and Revised Edition.

Crown 8vo., profusely illustrated, cloth gilt.

5/- net, 5/4 post free.

**NURSING: ITS THEORY AND PRACTICE.**

Being a complete Text-book of Medical, Surgical, and Monthly Nursing.

By Percy C. Lewis, M.D., M.R.C.S., L.S.A.

New Edition, Enlarged and Revised (17th thousand), profusely illustrated with over 100 new Cuts. Crown 8vo., cloth gilt.

3/6 post free.

**SURGICAL WARD-WORK.**

**SURGICAL WARD-WORK AND NURSING.**

By Alexander Miles, M.D.Edin., C.M., F.R.C.S.E.

Second Edition, Enlarged and entirely Revised.

Demy 8vo., copiously illustrated, cloth gilt.

3/6 net, 3/10 post free.

**ANATOMY.**

**ELEMENTARY ANATOMY AND SURGERY FOR NURSES.**

By William McAdam Eccles, M.S.Lond., M.B., F.R.C.S., L.R.C.P.

Crown 8vo., profusely illustrated, Cloth. 2/6 post free.

**PHYSIOLOGY.**

**ELEMENTARY PHYSIOLOGY FOR NURSES.**

By C. F. Marshall, M.D., B.Sc., F.R.C.S.

Crown 8vo., illustrated.

Cloth. 2/- post free.

**MASSAGE.**

**ART OF MASSAGE.**

By Mrs. Creighton Hale.

Second Edition.

Demy 8vo., 150 pp., profusely illustrated with over 70 Special Cuts.

Cloth gilt,

6/- post free.

**MIDWIFERY.**

**A PRACTICAL HANDBOOK OF MIDWIFERY.**

By Francis W. Haultain, M.D.

Now Ready.

New and Revised Edition.

Profusely illustrated with Original Cuts, Tables, &c.

Handsomely bound in leather.

6/- net, 6/3 post free.

*COMPLETE CATALOGUE POST FREE ON APPLICATION.*

*Of all Booksellers, or of*

THE SCIENTIFIC PRESS, LTD., 28 & 29 Southampton Street, Strand, London, W.C.

